# Medical Colleges

**Published:** 1874-04-15

**Authors:** 


MEDICAL COLLEGES.—"The past season has been a very successful one with our medical schools, the Jefferson graduating 151 men, and the University 121. Considering the great disadvantages the latter institution labored under, in being, as it were, houseless, and dwelling in the tents of a strange people, we think both the faculties are to be congratulated on their success. We do not doubt that the standard of graduation of these schools is as high as that of any similar institution in the country, always excepting noble Harvard; and the opportunities they offer for clinical study are almost unrivalled.

"If any student has the nerve and muscle to contend with New England climate, customs, and examination, and desires to get the most valuable diploma in the country, Harvard should be the school of his choice, and Boston his wintering city. To those of not such robust faith, purpose, and ability, we can heartily commend Philadelphia and its colleges." —*Philadelphia Medical Times.*

We copy the above paragraph for the purpose of both endorsing the compliment paid to Harvard, and of claiming equal title to the same for the Chicago Medical College, the medical department of the North-Western University of this city.

After a careful examination of the system adopted by the medical department of Harvard, during the last three years, we fail to discover anything, either in the fullness of its curriculum, its arrangement into three consecutive annual courses, the length of the college term, the frequency and thoroughness of the examinations, or in the requirements for the degree, that is different in principle, or more perfect in detail, than in the Chicago College.

The principal differences are, that the latter school has been successfully carrying out the system for fifteen years, instead of three; and the gradation of classes is enforced on all its students, while Harvard leaves it optional with hers.

